# From the Outside Looking in: Psoriasiform Dermatitis Presenting as a Paraneoplastic Syndrome for Pancreatic Adenocarcinoma

**DOI:** 10.7759/cureus.9067

**Published:** 2020-07-08

**Authors:** Shruthi Narasimha, Jalak Shah, Awa Drame

**Affiliations:** 1 Internal Medicine, University of South Florida Morsani College of Medicine, Tampa, USA

**Keywords:** pancreatic malignancy, paraneoplastic syndromes

## Abstract

Paraneoplastic syndromes exist for a number of malignancies and their presentations are diverse. Some of them are self-limited, while others can be life-threatening but regardless of the outcome, understanding the presentation is vital to picking up on the diagnosis for further treatment. The case that is presented here is a rare paraneoplastic skin process called Bazex syndrome that was associated with pancreatic adenocarcinoma.

## Introduction

Cancer is a devastating finding, but diagnosis can be difficult due to the vague symptoms and often indolent disease course. Sometimes, physical exam findings can serve as clues to possible malignancy precursors, but it can easily be overlooked when they are presented in different clinical pictures. Paraneoplastic syndromes can affect every organ system but the case discussed here deals with an extremely rare cutaneous manifestation known as acrokeratosis paraneoplastica or Bazex syndrome. First recognized in 1965, this syndrome presents with well-defined symmetrical scaly plaques. It is most often associated with squamous cell carcinomas (SCC) of the head and neck, but it has been documented less frequently in a number of other malignancies. This psoriasiform eruption is clinically relevant as it manifests months prior to the actual diagnosis of cancer. Astute clinicians who notice the signs and symptoms may be better able to work-up patients and earlier diagnosis may lead to more favorable disease outcomes [1,2].

This abstract was presented in October 2019 at the American College of Gastroenterology Conference.

## Case presentation

**Figure 1:**
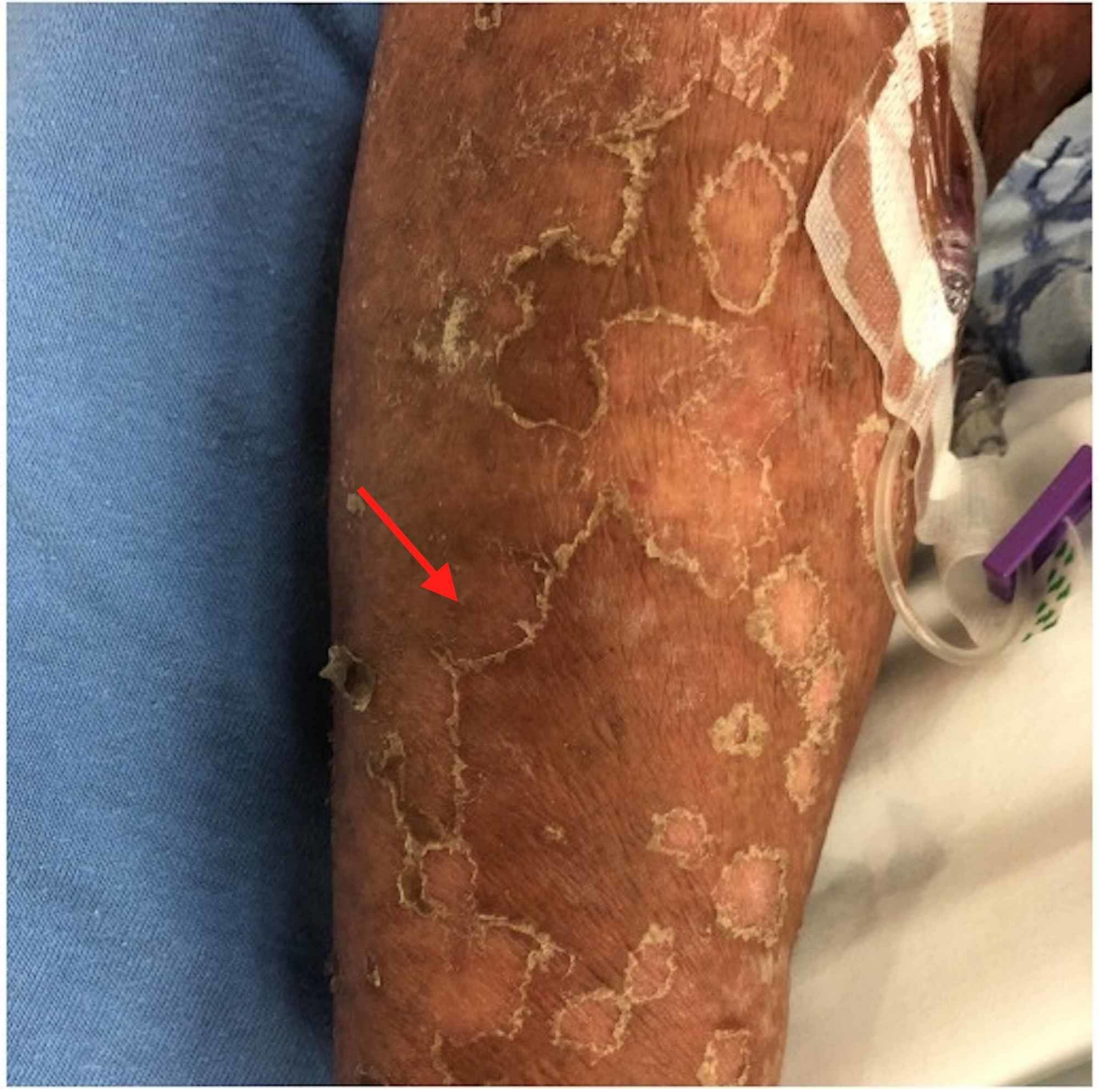
Psoriasiform dermatitis on upper extremity

**Figure 2:**
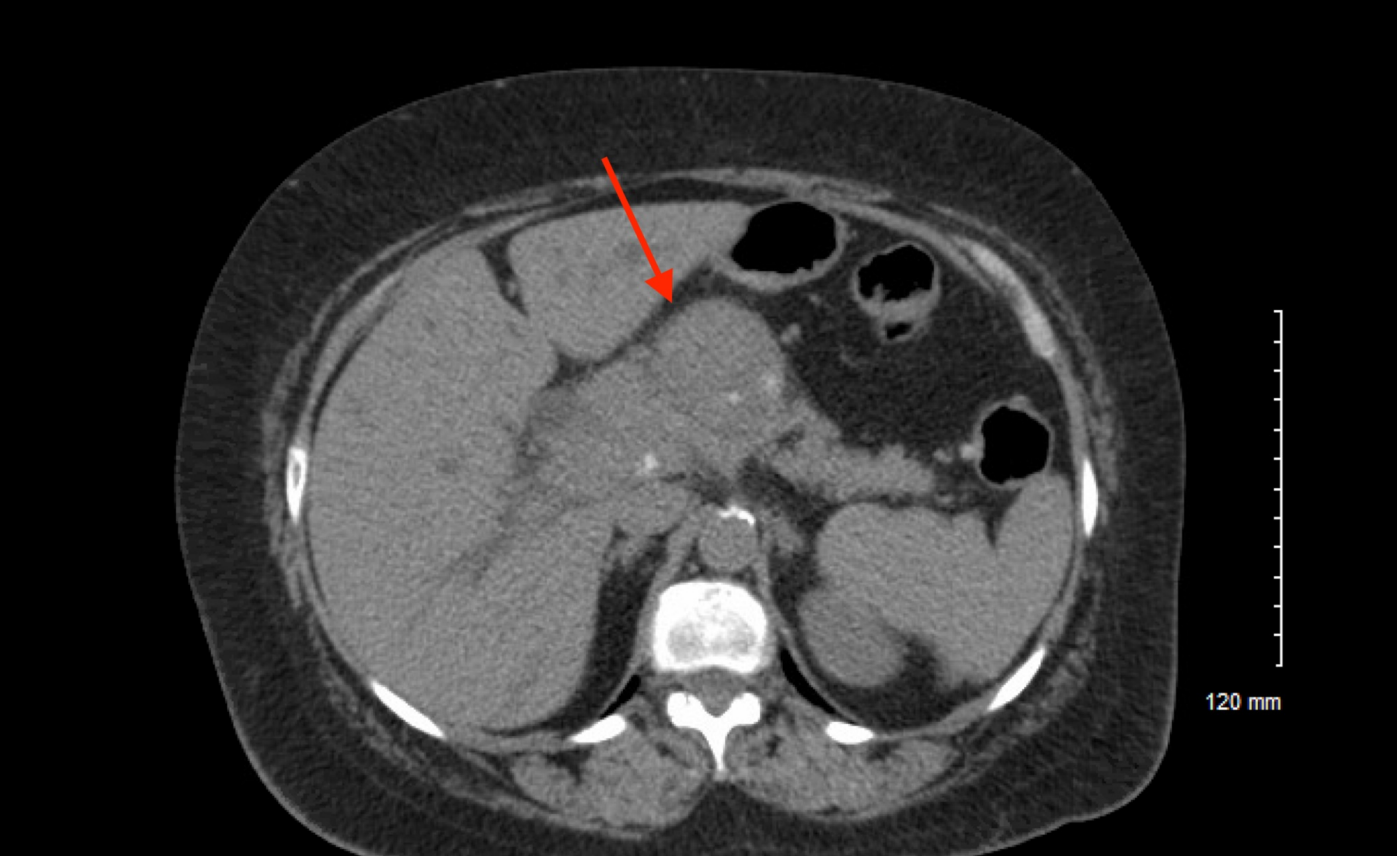
Several round pancreatic masses in the head and neck that encase the periportal structures Mass effect with the 4.1 x 4.5 cm pancreatic lesion noted and common bile duct is obstructed and displaced

This case involved a 64-year-old female with a history of hypertension and paroxysmal atrial fibrillation who came to the hospital with complaints of yellow discoloration of her skin, intermittent fevers and a recent history of right upper quadrant pain with nausea. Her primary care provider sent her to the emergency room after basic lab work revealed a total bilirubin of 14.3. Of note, the patient was previously seen in the hospital six months prior for a skin rash on her extremities and abdomen that was biopsied and later described as psoriasiform dermatitis (Figure 1). It was thought to be a drug reaction from a medication, but the rash still persisted after discontinuation. On this admission, a CT abdomen was ordered in the setting of painless jaundice and it revealed masses in the pancreatic head and neck with confluent disease encasing the periportal structures and was highly suspicious for malignancy (Figure 2). Biopsy after the endoscopic ultrasound was consistent with pancreatic adenocarcinoma. The patient was evaluated by gastroenterology and she had a biliary sphincterotomy with stenting to assist with the hyperbilirubinemia secondary to the pancreatic obstruction. Oncology staged her disease and started her on modified FOLFIRINOX therapy and she was discharged for further treatment and follow-up.

## Discussion

Bazex syndrome is a rare skin finding that has been associated with head and neck SCC, but it has rarely been seen in gastrointestinal malignancies. The cutaneous manifestations can include nail dystrophy, hyperpigmentation, violaceous papulosquamous lesions, and keratoderma with much less common bullae and dactylitis findings [2]. Physical signs and symptoms are broad but pathology will reveal psoriasiform dermatitis and it is usually resistant to all topical treatments. 60%-75% of patients present with the paraneoplastic process prior to known diagnosis and about 15%-25% have manifestations at the time of diagnosis. Treatment of the underlying malignancy has been shown to improve or eliminate lesions in 90%-95% of affected individuals. The underlying mechanism is unclear, but a number of theories exist. Immune-mediated reaction between cutaneous and tumor antigens, increased production of keratinocyte growth factors like TGH-alpha and co-existence of other bullous diseases processes are just a few explanations to explain this phenomenon [2,3].

## Conclusions

This case highlights a rare psoriasiform dermatitis rash associated with malignancies but more importantly, this paraneoplastic cutaneous process manifested prior to diagnosis. With more than 50% of patients presenting with cutaneous signs well before diagnosis, educated physicians can have a higher index of suspicion for malignancy, which can prompt earlier diagnosis and treatment to reduce morbidity and mortality.
